# Using adaptive psychophysics to identify the neural network reset time in subsecond interval timing

**DOI:** 10.1007/s00221-021-06227-0

**Published:** 2021-09-28

**Authors:** Renata Sadibolova, Stella Sun, Devin B. Terhune

**Affiliations:** Department of Psychology, Goldsmiths, University of London, New Cross, London, SE146NW UK

**Keywords:** State-dependent network, Breakpoint, Time perception, Temporal discrimination, Adaptive psychophysics

## Abstract

State-dependent network models of sub-second interval timing propose that duration is encoded in states of neuronal populations that need to reset prior to a novel timing operation to maintain optimal timing performance. Previous research has shown that the approximate boundary of this reset interval can be inferred by varying the inter-stimulus interval between two to-be-timed intervals. However, the estimated boundary of this reset interval is broad (250–500 ms) and remains under-specified with implications for the characteristics of state-dependent network dynamics sub-serving interval timing. Here, we probed the interval specificity of this reset boundary by manipulating the inter-stimulus interval between standard and comparison intervals in two sub-second auditory duration discrimination tasks (100 and 200 ms) and a control (pitch) discrimination task using adaptive psychophysics. We found that discrimination thresholds improved with the introduction of a 333 ms inter-stimulus interval relative to a 250 ms inter-stimulus interval in both duration discrimination tasks, but not in the control task. This effect corroborates previous findings of a breakpoint in the discrimination performance for sub-second stimulus interval pairs as a function of an incremental inter-stimulus delay but more precisely localizes the minimal inter-stimulus delay range. These results suggest that state-dependent networks sub-serving sub-second timing require approximately 250–333 ms for the network to reset to maintain optimal interval timing.

## Introduction

The human brain uses multiple systems to process with various degrees of precision temporal information spanning timescales over ten orders of magnitude (Buhusi and Meck 2005). Advances in our understanding of these timing mechanisms have been facilitated by studying these systems across a variety of stimulus ranges and contextual factors including emotional and attentional states, non-temporal stimulus properties (for a review, see van Wassenhove 2009) or the interval context arising from previous stimuli (Burr et al. 2013; Jazayeri and Shadlen 2010). An understudied issue in the temporal discrimination of interval pairs is when the context imposed by a stimulus interval ceases to affect the processing of a successive stimulus.

Temporal discrimination is widely used to index interval timing (Bausenhart et al. 2018). In temporal discrimination tasks, participants are typically presented with a pair of successive stimuli and asked to judge whether the second stimulus was longer or shorter compared to the first stimulus. The more similar the stimulus intervals are, the more difficult their discrimination becomes, which is reflected in a near-chance level discrimination performance. By contrast, the proportion of accurate responses will steadily increase when the stimulus intervals begin to noticeably differ. This “just-noticeable difference” proportional to the actual interval length is known as Weber’s fraction (WF). The WF that is constant across different interval lengths reflects the linear dependency of the noticeable difference on physical interval magnitudes according to Weber’s law (Gibbon 1977). A more common pattern, however, is larger WFs for brief intervals plateauing at longer intervals in accordance with a generalized form of Weber’s law based on a square root relationship (Burr et al. 2013; Lewis and Miall 2009). Both models predict that the physical difference between two long intervals must be larger than that between two brief intervals if the perceptual systems were to discriminate them as a pair of different interval lengths.

The temporal discrimination task has been extensively applied to investigate a range of factors modulating temporal cognition (Allman and Meck 2012; Benau and Atchley 2020; Oliveri et al. 2008; Wearden et al. 1998) and it has helped to generate novel insights into how our experience of time is formed and how the brain may represents time. For instance, the discrimination of very brief auditory stimuli is superior to that of the same intervals in the visual modality; however, this difference disappears once the interval length increases and more amodal higher-order cognitive resources are recruited (Rammsayer and Pichelmann 2018). These observations can be accounted for by models proposing that the timing of brief intervals (< 500 ms) is supported by an automatic, modality-specific timing system, whereas the timing of longer intervals is sub-served by a distinct cognitive timing mechanism (Gooch et al. 2011; Lewis and Miall 2003; Rammsayer and Pichelmann 2018). Although these models assume an interval changepoint that marks the transition between these systems, the characteristics, and perceptual and neural bases of this changepoint and these putative systems remain underspecified (Buhusi and Meck 2009; Gooch et al. 2011; Rammsayer and Pichelmann 2018).

The features of this transition are partly addressed by the state-dependent network (SDN) model (Buonomano and Merzenich 1998; Karmarkar and Buonomano 2007; Paton and Buonomano 2018), in which millisecond intervals are encoded in states of neuronal populations analogous to the evolving state of a liquid surface which has been disturbed by throwing in an object (Buonomano et al. 2009). One consequence of this model is that a network needs to dynamically reset to facilitate optimal timing: prior to network resetting, a timing operation will be deleteriously affected just as throwing in the second object before the liquid returns to its baseline state creates a distorted spatiotemporal pattern of the ripples on its surface. Previous studies suggest that neural networks supporting interval discrimination require between 250 and 500 ms to return to their initial state (Buonomano et al. 2009; Karmarkar and Buonomano 2007). Moreover, it has been suggested that a network may ‘time out’ and reset if the interval exceeds the maximum encodable length (~ 300 ms; Buonomano et al. 2009; Spencer et al. 2009).

In keeping with the premise that both the new stimulus interval and the ongoing network state (i.e., the context imposed by the previous interval) determine the response on a given trial, several psychophysical studies sought to assess the impact of preceding distractor intervals on temporal performance (Buonomano et al. 2009; Burr et al. 2013; Karmarkar and Buonomano 2007; Spencer et al. 2009). For example, Karmarkar and Buonomano (2007) applied a “reset task” consisting of interleaved trials with a single target interval bound by two tones and a distractor and target interval pair demarcated by three tones, rather than a standard temporal discrimination task. Congruent with the expectations of the SDN model, the target intervals of 100 ms, unlike those of 1000 ms, were characterized by poorer discrimination when preceded by distractor intervals. In another study (Spencer et al. 2009), the detrimental effect of a distractor on temporal discrimination was replicated for a 100 ms stimulus interval but was not observed when either the target or the distractor stimulus interval increased to 300 ms. One interpretation of these observations is that the network resets after a specific interval, i.e., at a maximum duration that the network might be capable of representing (Spencer et al. 2009).

It has been hypothesized that the boundary beyond which sub-second interval timing no longer relies on state-dependent computations can be identified as the inter-stimulus interval (ISI) between the target stimulus interval pair that is associated with an improvement in the temporal discrimination threshold (Buonomano et al. 2009). To evaluate this, Buonomano et al. (2009) had participants discriminate two auditory intervals (standard and comparison intervals), in blocks of trials distinguished by different ISIs (50, 250, 500, 750, and 1000 ms). The boundary for the putative reset interval was observed between 250 and 500 ms, as reflected by superior duration discrimination thresholds in longer ISI conditions.

The aim of this study was to build upon previous research (Buonomano et al. 2009; Karmarkar and Buonomano 2007) and more precisely identify the interval boundary of network resetting in the range of 250–500 ms. Toward this end, we measured duration discrimination thresholds for 100 ms and 200 ms standard stimulus intervals, and pitch discrimination thresholds as a control task, in conditions with different ISIs (range 250–583 ms). We expected a changepoint in duration discrimination thresholds across ascending ISIs that would generalize across the two interval conditions. This changepoint was hypothesized to reflect the boundary of the network reset for interval timing and thus was not expected in the control task.

## Methods

### Participants

Forty right-handed (Oldfield 1971) individuals participated in this study and 38 participants were included in the analyses after removing two multivariate outliers (82% female, 18% male, age range: 20–34; *M* = 25.63, SD = 3.66; years of higher education range: 0–8, *M* = 3.76; SD = 2.02 [four missing]). The sample size was determined a priori using G-power (v. 3.1.9.3; Faul et al. 2009) with a repeated-measures analysis of variance and parameters α = 0.05, 1-β = 80%, and η_p_^2^ = 0.08 (Buonomano et al. 2009), yielding a required sample size of *N* = 36. To account for attrition, we intended to include 40 participants and increased this to 41 when one participant, whose data were subsequently excluded, was unable to understand the tasks. All procedures were approved by the departmental Ethics committee at Goldsmiths, University of London.

### Materials

#### Duration and pitch discrimination tasks

**Fig. 1:**
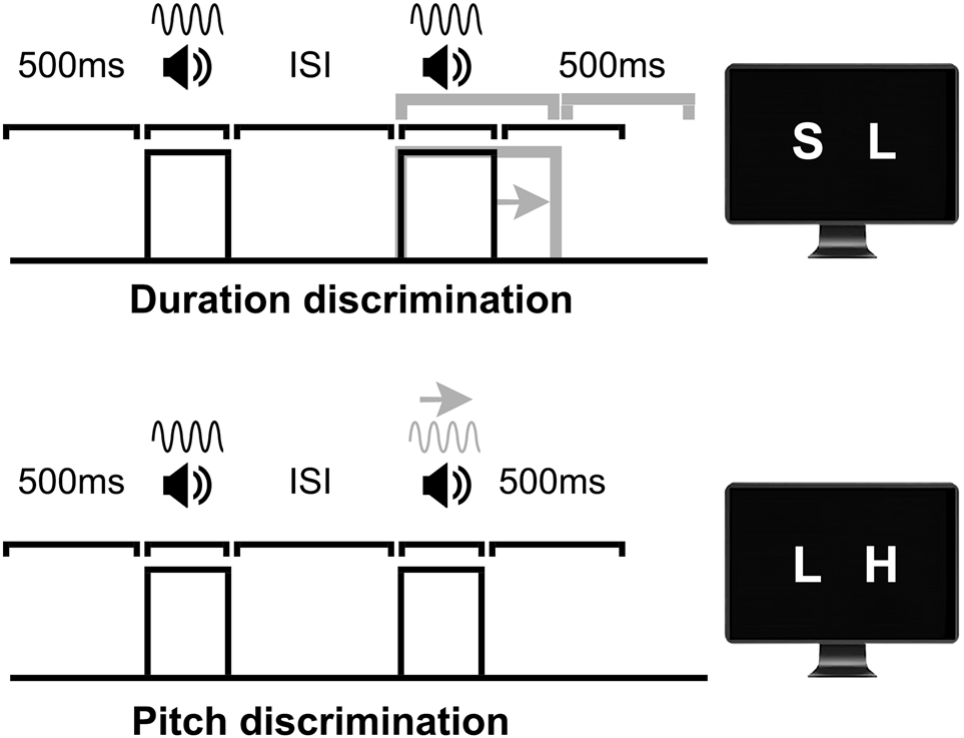
Diagrams of experimental tasks. All trials comprised a pre-stimulus interval (500 ms), a pair of tones separated by an ISI (250, 333, 417, 500 or 583 ms, varied at block level), a fixed post-stimulus interval (500 ms), and the response prompt. Participants estimated in three two-alternative forced-choice (2AFC) tasks if the second stimulus was shorter or longer (duration discrimination tasks) or lower or higher in pitch (pitch discrimination task) compared to the first stimulus. The standard stimulus (the first of two tones in the diagrams) was fixed in each task: 100 ms, 200 ms, and 1 kHz. The frequency for the duration standards was 1 kHz and the duration of the 1 kHz standard was 100 ms. The duration of the comparison stimulus (duration discrimination) and pitch of the comparison stimulus (pitch discrimination) were adaptively adjusted based on the performance on a trial-by-trial basis (gray arrows and lines). The standard-stimulus presentation order in the experiment was randomized within blocks

Participants completed two-duration discrimination tasks and a pitch discrimination task for the purpose of estimating discrimination thresholds as a function of ISI (250, 333, 417, 500 and 583 ms). In all tasks, the trial sequence consisted of a pre-stimulus interval (500 ms), a pair of tones separated by an ISI, a post-stimulus interval (500 ms), and a visual response prompt (Fig. 1). The two tones consisted of a fixed standard tone and a comparison tone that varied adaptively with performance, with order of presentation counterbalanced within blocks. At the prompt, participants judged whether the second tone was shorter or longer than the first tone (S L or L S [S = shorter; L = longer]) or lower or higher in pitch (L H or H L [L = lower; H = higher]). Participants responded with their right index and middle fingers on the left and right arrow keys of a keyboard, respectively, with the response-key mappings (S L vs. L S and L H vs. H L) counterbalanced across participants. Auditory stimuli were generated in MATLAB 2018b (MathWorks, Natick) in real-time using the PsychPortAudio function of Psychtoolbox-3 (Brainard 1997; Kleiner et al. 2007), using the Windows 7 WASAPI sound device. We sampled stereo sounds at 48 kHz default rate and presented them via the headphones at a constant intensity level (set to 0.01 programmatically and 70% in Windows sound settings).

In the two-duration discrimination tasks, the standard stimulus duration (*d*) was 100 ms and 200 ms, respectively, whereas the comparison stimulus lasted *d* + Δ*d*. In these two tasks, pitch remained constant (1 kHz). Analogously in the pitch discrimination task, the pitch for standard and comparison stimuli was, respectively, *p* = 1 kHz and *p* +  Δ*p*, and stimulus intervals were fixed at 100 ms. The change ( Δ) in duration or pitch was always a positive value and it was computed automatically on trial-by-trial basis as a psychometric threshold using a Bayesian adaptive staircase method (Ψ-marginal algorithm) implemented in the Palamedes toolbox for MATLAB (Kingdom and Prins 2016; Prins and Kingdom 2018). This method optimizes both the sampling and estimation of the target psychometric parameter(s), including participant’s responses into a prior distribution that affects subsequent values tested.

Our objective was to identify the  Δ for a reliably discriminable stimulus pair (threshold, alpha parameter), with a subsidiary assessment of temporal precision (beta) and attentional lapse rate (lambda) (Kingdom and Prins 2016). The guess rate (gamma) was fixed at 50%. The psychometric function parameters were updated after each trial response (correct vs. incorrect) and responses were fitted with a logistic Weibull function. The dependent measure was each block’s final discrimination threshold, i.e., the comparison stimulus Δ with 75% probability of a correct response. Each task (standard condition) comprised five blocks of 40 trials, one for each ISI. We constrained the prior beta and lambda parameters to values from zero to four in steps of 0.1 and zero to 0.2 in steps of 0.02, respectively. The initial prior alpha range was 100 to 300 ms and 200 to 400 ms (both in 1 ms steps), respectively, for the 100 ms and 200 ms standards and 1 kHz to 1.5 kHz (5 Hz steps) for the 1 kHz standard. These upper boundaries were extended for acceptable comparison stimulus range by 300 ms in duration discrimination tasks and 2 kHz for pitch discrimination.

### Procedure

Following general instructions, the experimenter confirmed with each participant that the stimulus volume was well above the detection threshold yet within a safe audible range. Prior to each task, participants completed ten practice trials with randomly selected standard-comparison stimulus pairs. Participants subsequently completed five consecutive blocks for each of the three tasks in randomized order (each corresponding to a unique ISI: 250, 333, 417, 500 and 583 ms) resulting in 15 blocks. Task order was counterbalanced across participants. To avoid fatigue, short breaks after each block were encouraged. The entire experiment took approximately 60 min.

### Analyses

**Fig. 2:**
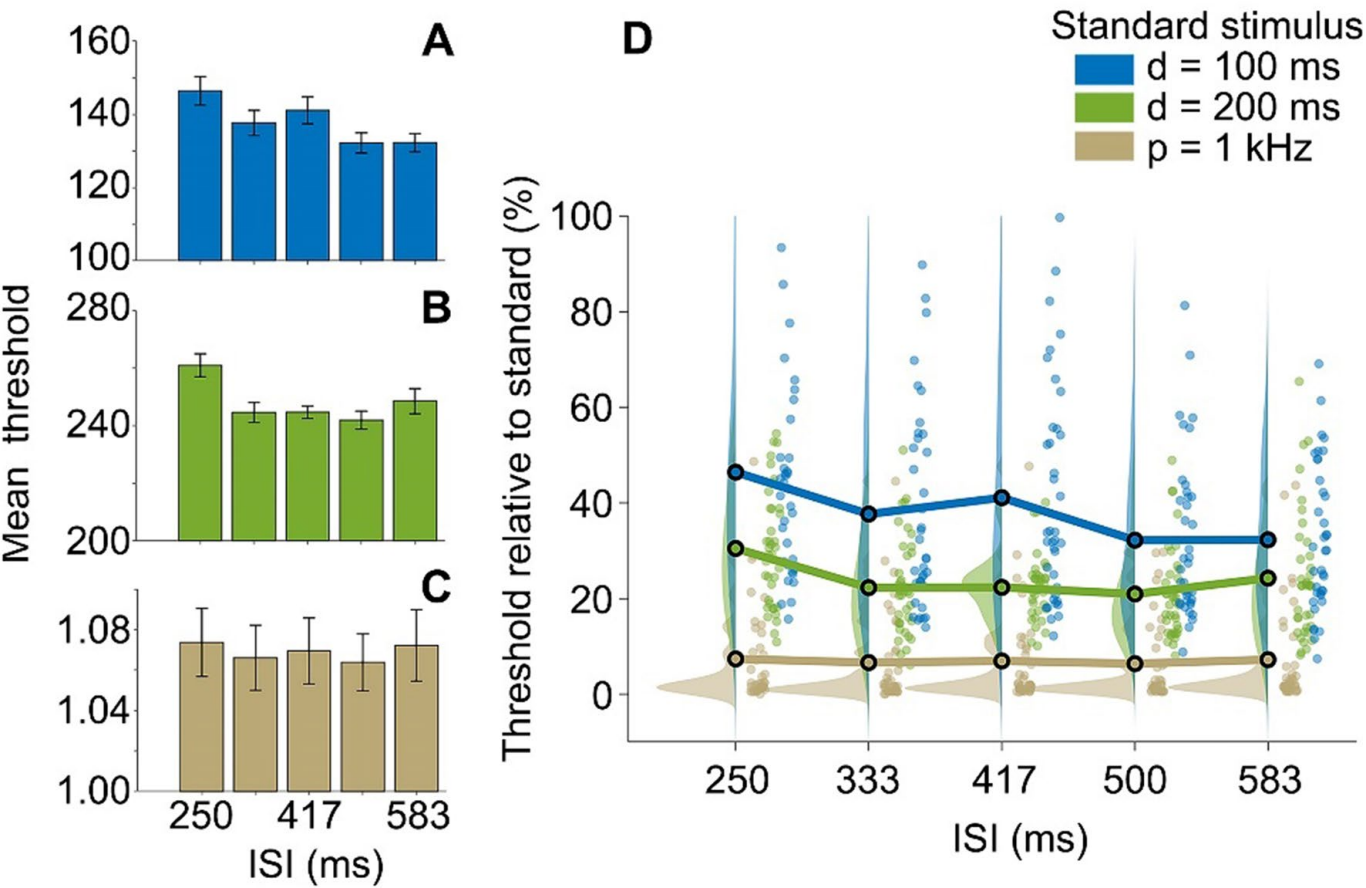
Duration (d) discrimination and pitch (p) discrimination as a function of the ISI (ms) between standard and comparison stimuli. **A–C** ∆ (75% discrimination threshold) for different ISIs in duration and pitch discrimination tasks. Error bars indicate standard error of the mean (SEM). **D** ∆ scaled by the respective standard stimulus. Marginal plots show the kernel density distributions and individual participant data in each condition (Allen et al. 2019)

Two participants were removed as multivariate outliers with Mahalanobis distance values > 31.02, *p* = 0.001. Data were reliably characterized by a departure from normality (Shapiro–Wilk test *p* < 0.05, Fig. 2D) including after transformations aimed at reducing positive skew (square root and log transform). Thus, they were analyzed using non-parametric Friedman tests and Wilcoxon signed-rank tests (in IBM SPSS Statistics software v.24). *P* values for the latter tests were adjusted using a Holm–Bonferroni multiple-comparison correction (Holm 1979). We report Kendal W (*r*_*t*_) effect size for the Friedman tests and *r* (*r* = *z/*√*N*; Pallant 2007) for the Wilcoxon tests. Bayes factors were not computed due to violations of normality (Dienes 2014; Rouder et al. 2012; Wetzels et al. 2012). In a series of complementary analyses, we additionally fitted exponential functions to discrimination thresholds across ascending ISIs in each condition and participant (using the fit command in MATLAB) and conducted a repeated-measures ANOVA on the exponential decay coefficients. Here, we also report partial eta-squared (*η*^*2*^) and Bayes factors (BF) which we computed using default priors in JASP software (JASP Team 2019). Additional one-sample *t* tests were conducted on exponential decays in each standard condition with *p* values adjusted using a Holm–Bonferroni correction.

## Results

Previous research suggests that duration discrimination varies according to the ISI between two intervals, such that a rapid succession (short ISI) is associated with poorer performance (higher discrimination threshold) (Buonomano et al. 2009). As can be seen in Fig. 2A, B, D, performance patterns on the duration discrimination tasks generally conformed to this pattern as the highest discrimination thresholds in both tasks were observed for the shortest ISI (250 ms). These effects were reflected in significant main effects of ISI on duration discrimination thresholds in both tasks with similar effect sizes, 100 ms: *χ*^*2*^_*F*_(4) = 25.50, *p* < 0.001, *r*_*t*_ = 0.17, and 200 ms: *χ*^*2*^_*F*_(4) = 20.78, *p* < 0.001, *r*_*t*_  = 0.14. As anticipated, a corresponding main effect of ISI was not observed for pitch discrimination thresholds, *χ*^*2*^_*F*_(4) = 3.87, *p* = 0.42, *r*_*t*_ = 0.03, with a near-zero effect size, showing relative uniformity across ISIs (Fig. 2C, D). These results suggest that duration discrimination thresholds selectively vary as a function of ISI.

To identify the ISI at which the earlier stimulus ceases to interfere, we conducted four planned comparisons of duration discrimination thresholds between the adjacent ISIs in each duration discrimination task. In the 100 ms standard task, thresholds were higher in the 250 ms (Mdn = 143.60 ms) than in the 333 ms (Mdn = 128.40) ISI condition, although this difference was only observed at a trend level, *Z* = − 2.47, *p* = 0.08, *r* = − 0.40 (Fig. 2A). There was no significant difference between thresholds in the 333 ms and 417 ms ISI conditions (Mdn = 133.00), *Z* = − 0.50, *p* = 1.00, *r* = − 0.08. By contrast, discrimination thresholds were significantly lower (improved) in the 500 ms (Mdn = 126.75) relative to the 417 ms ISI condition, *Z* = − 3.25, *p* = 0.01, *r* = − 0.53 and were not significantly different between the 500 and 583 ms (Mdn = 130.05) ISI conditions, *Z* = − 0.15, *p* = 1.00, *r* = − 0.02. This pattern of results replicates previous observations (Buonomano et al. 2009) and suggests that the network requires less than 500 ms to reset. However, there was some ambiguity regarding the precise window of this reset with weak evidence for an early reset (250–333 ms) and additional evidence for a later reset (417–500 ms). Such ambiguity was not present in the 200 ms standard task where there was clear evidence for an earlier boundary in alignment with the former effect. In particular, duration discrimination thresholds were significantly greater in the 250 ms (Mdn = 259.80 ms) than in the 333 ms (Mdn = 242.45 ms) ISI condition, *Z* = − 3.48, *p* = 0.01, *r* = − 0.56. Duration discrimination thresholds remained relatively stable across the remaining ISI conditions (Fig. 2B), 333 ms vs. 417 ms (Mdn = 244.75 ms), *Z* = − 0.66, *p* = 1.00, *r* = − 0.11, 417 ms vs. 500 ms (Mdn = 237.90 ms), *Z* = − 1.29, *p* = 1.00, *r* = − 0.21, and 500 ms vs. 583 ms (Mdn = 245.50 ms), *Z* = − 1.31, *p* = 1.00, *r* = − 0.21. Cumulatively, these results suggest a boundary for this network reset between 250 and 333 ms.

Duration discrimination thresholds scaled by the standard stimulus (Fig. 2D) were higher in the 100 ms (Mdn = 34.85%) compared to the 200 ms standard stimulus condition (Mdn = 23.97%), *Z* = − 5.36, *p* < 0.001, *r* = − 0.87. Although they would be expected to be similar according to the scalar property or Weber’s law (Gibbon 1977), past research has demonstrated that Weber’s law in its strict form often does not hold (Grondin 1993, 2012). Our finding conforms to a common pattern in the literature showing higher WFs for very brief to-be-discriminated intervals, which is consistent with a generalized form of Weber’s law (Burr et al. 2013; Lewis and Miall 2009). To explore the interaction of the ISI and standard stimulus conditions, particularly the later reset for the 100 ms vs. the 200 ms standard condition, we subtracted the scaled thresholds in the 100 ms condition from those in the 200 ms condition. A Friedman test on the difference in scaled thresholds did not yield a significant effect, *χ*^*2*^_*F*_(4) = 6.80, *p* = 0.15, *r*_*t*_ = 0.05. These results suggest that the two standard stimulus conditions did not significantly differ in the reset interval.

**Fig. 3:**
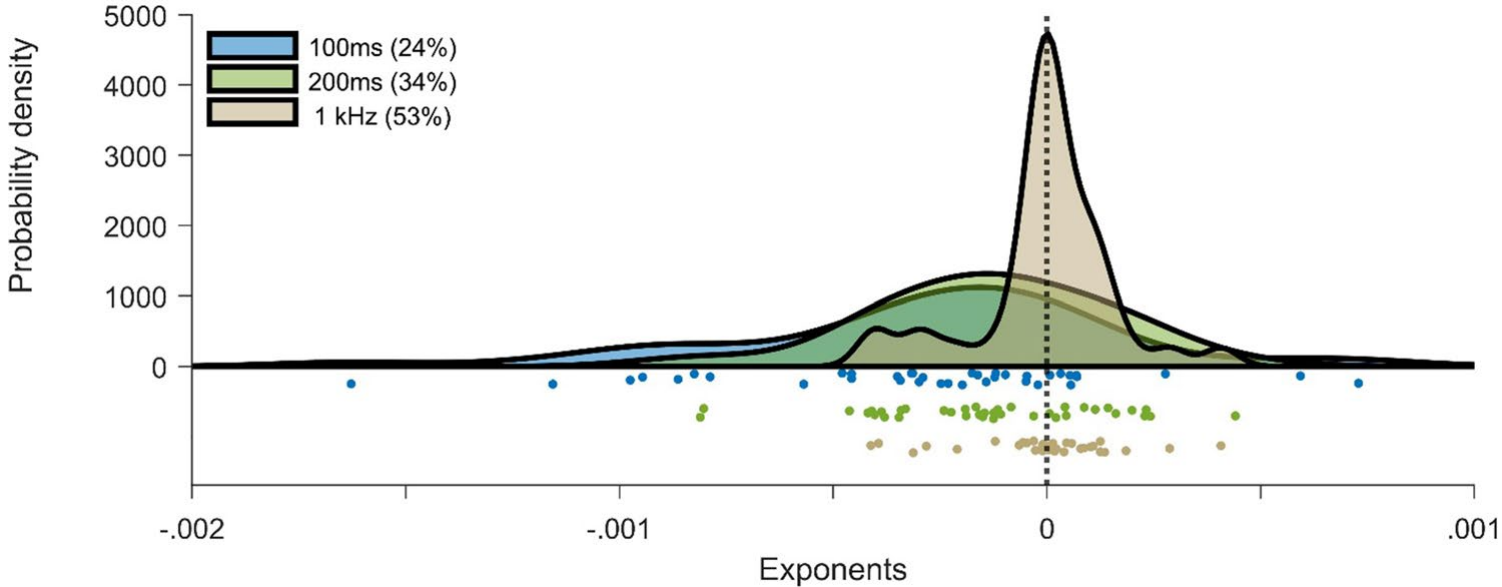
Exponents of the exponential decay function fitted to discrimination thresholds across the ISIs in each condition (*N* = 38). The plot shows kernel density distributions and data of individual participants in each condition. Bracketed values indicate the proportion of participants with exponents > 0 in each condition

A similar conclusion was reached in the analysis of slopes of the exponential decay function fitted to thresholds across ascending ISIs for each participant and standard condition. The exponents were used to evaluate how individual participants conformed to overall group-level trends (Fig. 3). We observed negative exponents reflecting the reduction in discrimination thresholds from 333 ms onwards in the 100 ms and 200 ms standard conditions, one-sample *t*(37) = 3.85, *p* = 0.001, *d*_*z*_ = 0.62, BF_10_ = 62.17 and *t*(37) = 3.08, *p* = 0.010, *d*_*z*_ = 0.50, BF_10_ = 9.29, respectively, but not in the 1 kHz condition, *t*(37) = 0.16, *p* = 0.87, *d*_*z*_ = 0.03., BF_10_ = 0.18. An ANOVA showed that exponential decay differed across standard conditions, *F*(2,74) = 7.19, *p* = 0.003, *η*^*2*^ = 0.16. Subsequent post hoc analyses further showed a more pronounced decays in the 100 ms vs. 1 kHz, *t*(37) = 3.48, *p* = 0.004, *d*_*z*_ = 0.56, BF_10_ = 24.24, and 200 ms vs. 1 kHz conditions, *t*(37) = 2.58, *p* = 0.04, *d*_*z*_ = 0.42, BF_10_ = 3.11. Although the difference between 100 and 200 ms conditions was non-significant, the Bayesian evidence trending in favor of the null hypothesis was ambiguous, *t*(37) = 1.74, *p* = 0.27, *d*_*z*_ = 0.28, BF_10_ = 0.68.

## Discussion

SDN models of interval timing propose that optimal sub-second interval timing requires resetting of neuronal networks encoding stimulus duration prior to a new timing operation (Karmarkar and Buonomano 2007; Paton and Buonomano 2018; Spencer et al. 2009). This study sought to estimate the putative network reset interval by varying the ISI between comparison and standard intervals in a sub-second auditory interval discrimination task (Buonomano et al. 2009). We found that interval discrimination thresholds significantly improved (decreased) in the 200 ms standard condition and suggestively improved in the 100 ms standard condition when the ISI increased from 250 to 333 ms, with moderate effect sizes in both cases. By contrast, analogous comparisons for pitch discrimination thresholds yielded non-significant results. These findings are consistent with previous research (Buonomano et al. 2009; Karmarkar and Buonomano 2007) suggesting that the network reset interval is between 250 and 500 ms, warranting further research on the characteristics and dynamics of network resetting in sub-second interval timing.

Computational studies of SDNs have conventionally included a constraint that the physiological mechanisms are of limited temporal extent after which the network resets (Buonomano and Merzenich 1998; Karmarkar and Buonomano 2007). Germane behavioral evidence suggests that SDNs sub-serving interval timing reset between 250 and 500 ms (Buonomano et al. 2009). This inference was made on the basis of a decrease in discrimination thresholds for pairs of intervals separated by 500 ms, relative to 250 ms ISIs. Our results corroborate this time window for the putative SDN reset (250–500 ms) and show that this effect generalizes to a 200 ms standard interval stimulus condition. Moreover, we further expanded upon previous findings through the inclusion of a greater number of ISIs during the putative breakpoint window (250, 333, 417, 500 ms) to permit greater precision in the estimate of the network reset interval. As a result, we identified a narrower reset time window of 250–333 ms which aligns with the observation of improved temporal performance in a ‘reset task’ when either the distractor interval or immediately following *target* interval increased from 100 to 300 ms (Spencer et al. 2009).

Although the reset time, and therefore the inability to accommodate longer intervals, has previously been considered to be a limitation of the applicability of the SDN model in timing (Spencer et al. 2009), the presence of a mechanism dedicated to the processing of sub-second intervals is congruent with more recent advances in the interval timing literature. For instance, Rammsayer and Pichelmann (2018) introduced a conceptual model of sub and supra-second timing, arguing for distinct modality-specific neurocognitive mechanisms sub-serving the timing of brief intervals (below ~ 100–500 ms) that gradually gives way to amodal mechanisms responsible for the processing of longer intervals. Indeed, the time-dependent changes in the state of neural networks are likely to incorporate modality-specific neural codes for brief intervals but much less so for longer intervals recruiting executive functions (Paton and Buonomano 2018). Numerous studies, reporting psychopharmacological (Rammsayer 1993; Rammsayer and Vogel 1992), psychophysical (Karmarkar and Buonomano 2007; Rammsayer et al. 2015), neuroimaging (Lewis and Miall 2003; Wiener et al. 2010) or genetic (Wiener et al. 2011) evidence, add further weight to the argument of distinct timing mechanisms in the millisecond-to-second range. However, the boundary between these putative timing systems, and the nature of a transition between them, remains controversial. Our findings would suggest that the boundary between sub-second timing systems is within this 250–333 ms window.

Although the present results provide a more refined estimate of the network reset interval window than previous research, the characteristics of this reset interval require further specification. Further research is required to more precisely delineate the window of this reset interval. In particular, further research would benefit from adaptively varying the ISI between standard and comparison intervals to derive a more precise estimate of the network reset interval. The present work suggests that this interval will be observed between 250 and 333 ms. It will also be important to determine whether this reset interval generalizes across sensory modalities or co-varies with superior temporal precision in auditory relative to visual timing (Penney et al. 2000). A further outstanding question concerns the role of the network reset interval in differentiating timing mechanisms for sub-second and supra-second intervals. Our evidence suggests that the breakpoint that co-occurs with the network reset interval is specific to sub-second interval timing. Nevertheless, it remains understudied whether the shift to different mechanism for longer intervals is abrupt or gradual. Further work would therefore benefit from using the present approach to probe the division between sub-second and supra-second timing.

Discriminated intervals are not only timed, but obviously subject to non-specific attentional, memory and decisional influences that shape behavioral responses (Brown 1997; Droit-Volet and Meck 2007; Grondin 2010; Toren et al. 2020). Whereas the SDN model is attractive given its plausible neurobiological basis and our data conform to its basic conceptualization, further work is required to more precisely interrogate its predictions against alternative constraints on timing. For instance, short ISIs may interact with task difficulty and interfere with encoding of the first stimulus due to a prompt presentation of the second interval of a stimulus pair. This would in turn affect the implicit learning that may improve the precision of neural timers during the repeated task exposure. Anecdotal evidence is offered in studies of children learning a language that is enhanced by an exaggeration of empty intervals between words (i.e., “Motherese”; Broesch and Bryant 2015). The same principle applies in learning of the Morse code when letters represented by dots and lines are transmitted at normal speed but the pauses between the letters and words are emphasized (Bloom 1990; Buonomano 2017).

In conclusion, previous research suggests that interval timing engages disparate neural mechanisms, depending on the timescale and computational requirements of the task (Paton and Buonomano 2018). The SDN model represents a category of intrinsic timing models implicated in the encoding of brief sub-second intervals by means of trajectories in neuronal space. Whereas further research is required to disentangle the contributions of different timing mechanisms, our results build upon previous research (Buonomano et al. 2009; Karmarkar and Buonomano 2007) and provide a more precise estimate of the temporal window of the SDN reset and suggest that approximately 250–333 ms is required for the network to reset to facilitate optimal interval timing.

## Data Availability

The data and materials are available at https://osf.io/e6w79/.

## Code availability

Custom scripts are available at https://osf.io/e6w79/.
